# Lexical Diffusion: Linguistic Characteristics of Ophthalmology Science Articles with Disclosed Artificial Intelligence-Assisted Writing

**DOI:** 10.1016/j.xops.2026.101418

**Published:** 2026-09-21

**Authors:** Taiga Inooka, Kenya Yuki, Koji M. Nishiguchi

**Affiliations:** Department of Ophthalmology, Nagoya University Graduate School of Medicine, Nagoya, Japan

**Keywords:** Generative artificial intelligence, Large language models, Scientific writing, Ophthalmology

## Abstract

**Objective:**

To characterize discussion-section language in ophthalmology research articles with disclosed generative artificial intelligence (AI) writing assistance and to examine temporal lexical patterns among articles without disclosure.

**Design:**

Cross-sectional observational study.

**Subjects:**

Original research articles published in *Ophthalmology Science* from January 2024 through May 2026.

**Methods:**

Articles were classified according to disclosure of generative AI writing assistance. Discussion-section text was analyzed for Flesch Reading Ease (FRE), Flesch–Kincaid Grade Level (FKGL), Simple Measure of Gobbledygook (SMOG), predefined generative AI–associated terms, control academic terms, and their ratio. Articles with disclosed AI writing assistance were compared with nondisclosing articles using repeated stratified 1:5 resampling by publication year and first-author English-language–dominant affiliation status across 1000 iterations. Temporal trends were evaluated among nondisclosing articles.

**Main Outcome Measures:**

Flesch Reading Ease, FKGL, SMOG, term densities, and AI-associated-to-control term ratio.

**Results:**

Among 633 articles, 28 (4.4%) disclosed generative AI writing assistance. English-language–dominant affiliation fractions were lower among AI-disclosed articles for first and corresponding authors (*P* = 0.008 and 0.012, respectively). Artificial intelligence–disclosed articles had lower FRE (11.97 vs. 21.59), higher FKGL (17.15 vs. 16.00), and higher SMOG scores (17.82 vs. 17.12; *P* values < 0.001, 0.013, and 0.027, respectively). Resampling produced mean Hodges–Lehmann shifts of –7.35 for FRE (95% resampling interval, –8.90 to –5.83), 1.06 for FKGL (0.80–1.32), and 0.66 for SMOG (0.47–0.86). Artificial intelligence–associated term density and the AI-associated-to-control term ratio were higher in AI-disclosed articles (22.38 vs. 15.71 terms per 1000 words and 0.91 vs. 0.63; *P* = 0.003 and 0.004, respectively), whereas control-term density did not differ (*P* = 0.844); resampling confirmed these lexical findings. Among nondisclosing articles, AI-associated term density and the ratio increased over publication years (all, *P* < 0.001), whereas control-term density did not (all, *P* > 0.05).

**Conclusions:**

Disclosed generative AI writing assistance was uncommon but was associated with lower formula-based readability, higher estimated reading grade levels, and a higher density of predefined generative AI–associated vocabulary in discussion sections. The temporal pattern among nondisclosing articles also suggests possible corpus-level diffusion of AI-associated lexical patterns, although disclosure status does not verify actual AI use.

**Financial Disclosure(s):**

Proprietary or commercial disclosure may be found in the Footnotes and Disclosures at the end of this article.

Recent advances in large language models (LLMs) have spurred interest in ophthalmology for clinical^1^, ^2^, ^3^ and educational applications.^4^^,^^5^ Beyond these uses, generative artificial intelligence (AI) tools have also been incorporated into scientific writing, including drafting, language editing, and translation in academic manuscripts.^6^^,^^7^ Their growing use has prompted editorial and ethical discussions, and many journals now require transparent disclosure of LLM assistance.^8^ In ophthalmology, journal-level analyses have documented the evolving landscape of generative AI disclosure guidance.^9^^,^^10^

Despite these policy developments, it remains unclear whether disclosed generative AI writing assistance is associated with measurable differences in the language of published scientific articles. Prior studies have largely relied on indirect approaches, including temporal analyses of vocabulary shifts in the post-ChatGPT era^11^^,^^12^ and evaluations of AI-generated text detection tools.^13^ Although such approaches provide important corpus-level insights, they typically depend on temporal proxies or algorithmic classification rather than article-level disclosure. In addition, prior work has often focused on abstracts or broad publication-level trends, leaving full-text sections—particularly the discussion, where interpretation, framing, and narrative structure are most prominent—less directly examined.

This study aimed to characterize discussion-section language in ophthalmology research articles with disclosed generative AI writing assistance. Using formal declaration statements of generative AI and AI-assisted technologies in the writing process as a disclosure-based anchor, articles with and without such declarations were compared in a single ophthalmology journal. Complementary dimensions of writing were evaluated, including readability and predefined generative AI–associated lexical patterns with control academic terms, and associations between disclosure and authorship characteristics, such as first- and corresponding-author affiliation with English-language–dominant countries, were examined. Temporal trends in generative AI–associated lexical patterns among articles without disclosed AI writing assistance were also examined. These analyses may help clarify how generative AI writing assistance and AI-associated language patterns are emerging within ophthalmic scientific writing and may inform ongoing discussions regarding transparency, disclosure, and editorial policy.

## Methods

### Study Design

This cross-sectional observational study analyzed publicly available published articles. This study did not involve human participants or patient-level data; therefore, institutional review board approval was not required.

### Data Source

Original research articles published in *Ophthalmology Science* from volume 4, issue 1 (January–February 2024) through volume 6, issue 5 (May 2026) were identified through the journal’s publicly accessible online archive. Only articles listed under the “original articles” heading in each issue’s online table of contents were included; articles listed under other headings, including Editorial, Reports, Case Reports, Correspondence, and Errata and Corrigenda, were excluded. Systematic reviews and meta-analyses were included when listed under “original articles.” All included articles were freely available in full text at the time of data collection on May 9, 2026. Full-text article information was extracted from publicly available article pages, PDFs, and PubMed Central versions when available. One article without disclosed generative AI writing assistance was excluded from the linguistic analysis because it used an atypical topic-based narrative structure without a discrete discussion section, precluding consistent isolation of discussion-section text. The final analytic data set consisted of 633 original research articles. During the publication period of the included articles, Elsevier had already issued author guidance regarding the use of AI and LLMs, including recommendations for transparent disclosure of generative AI assistance (Elsevier AI Author Guide, 2023; https://authors.org.nz/wp-content/uploads/2023/06/Elsevier-AI-Author-Guide.pdf, accessed on February 5, 2026). This guidance indicates that declaration statements of generative AI and AI-assisted technologies in the writing process were expected when applicable.

### Identification of Generative AI Disclosure

Articles were classified as having disclosed generative AI writing assistance only when a declaration indicated that generative AI or AI-assisted tools were used for manuscript preparation, writing, editing, language improvement, readability improvement, grammar correction, or related text-preparation purposes. Articles without such declarations were classified as having no disclosed AI writing assistance. Declarations indicating that AI was not used for manuscript preparation, or describing generative AI use solely as part of the research methodology rather than manuscript writing, were also classified as having no disclosed AI writing assistance. Because the publisher’s declaration category includes both generative AI and AI-assisted writing technologies, the exposure was defined by disclosure of writing-related AI-assisted technology rather than by verified LLM use.

### Authorship Characteristics and English-Language–Dominant Affiliation Fractions

Authorship information was extracted from each article, including first authors, corresponding authors, author-affiliation mappings, and institutional affiliations. Countries of affiliation were identified through standardized parsing and normalization of affiliation text. English-language–dominant countries were defined as the United States, United Kingdom, Canada, Australia, New Zealand, and Ireland. This classification reflects institutional language context rather than individual native language, English proficiency, or actual manuscript drafting responsibility. For each article, English-language–dominant affiliation exposure was calculated separately for first authors and corresponding authors using fractional weighting. When multiple cofirst or cocorresponding authors were present, each author contributed equally. When an author had multiple affiliations, affiliations contributed equally within that author. The resulting article-level fraction ranged from 0 to 1, with 0 indicating no English-language–dominant affiliation among the relevant author group and 1 indicating exclusively English-language–dominant affiliation. A binary variable, defined as a fractional value of ≥0.5, was used only for stratified resampling analyses. This threshold was used because first-author fractions were concentrated at 0 and 1; joint stratification by publication year and exact fractional values would have produced sparse strata for repeated 1:5 resampling.

### Text Processing and Linguistic Measures

The discussion section was selected a priori as the primary text for linguistic analysis because it represents an interpretive and narrative component of scientific articles. For eligible original articles, section boundaries were identified using standardized headings when available; rule-based text parsing was applied when an explicit discussion heading was absent. Non-narrative elements, including references, tables, figure legends, acknowledgments, financial disclosures, and supplementary materials, were excluded. Only narrative prose within the discussion section was retained. Extracted discussion-section text was manually reviewed for all articles with disclosed AI writing assistance and for a random sample of articles without disclosed AI writing assistance to confirm section boundaries and exclusion of non-narrative material.

Readability was quantified using Flesch Reading Ease (FRE), Flesch–Kincaid Grade Level (FKGL), and the Simple Measure of Gobbledygook (SMOG) index. Lower FRE scores indicate lower readability, whereas higher FKGL and SMOG scores indicate higher estimated reading grade levels. Underlying components included discussion word count, mean sentence length (word count divided by sentence count), mean syllables per word (syllable count divided by word count), and polysyllabic words (≥3 syllables), with polysyllabic words reported both as an absolute count and per 100 words. Calculations used textstat version 0.7.13 with the language explicitly set to en_US; word counts were generated using lexicon_count with punctuation removal enabled, sentence and syllable counts using sentence_count and syllable_count, and polysyllabic-word counts using polysyllabcount. Under this setting, textstat uses entries from the Carnegie Mellon University Pronouncing Dictionary when available and a Pyphen-based fallback for words not found in the dictionary. No ophthalmology-specific syllable dictionary or manual term corrections were applied.

For dictionary-based lexical analysis, a predefined lexical dictionary was reconstructed from a previously published study^12^ that identified terms frequently used by LLMs or reported to have increased in frequency after the emergence of ChatGPT. The source study described these items as potentially AI-influenced terms; in the present study, they are referred to as predefined generative AI–associated terms to avoid implying article-level causality. The predefined dictionary was not intended to classify individual manuscripts as AI-generated or human-written, but rather to quantify corpus-level lexical patterns associated with previously reported AI-associated vocabulary. To align with the original search strategy, all explicitly specified terms within “…”[Text Word] clauses in the published appendix were extracted. This yielded 296 generative AI–associated word forms or phrases and 184 control academic word forms or phrases, with no overlap between the 2 dictionaries. The appendix-based extraction results also confirmed 135 representative AI-associated terms and 84 representative control terms before expansion into explicitly specified word forms. The control terms in the source study were common academic words or short phrases selected from lexical bundles previously identified in medical research, after excluding expressions overlapping with the AI-associated set. No matching of the control and AI-associated term sets by baseline frequency, topic neutrality, or part-of-speech distribution was reported in the source study, and no additional matching was performed in the present analysis. Text preprocessing included conversion to lowercase and whitespace normalization. Terms explicitly referring to generative AI itself, including terms such as “ChatGPT,” “prompt,” “large language model,” “LLM,” “generative AI,” and “artificial intelligence,” were removed before dictionary scoring. Dictionary terms were matched using regular expressions with word-boundary anchors to avoid partial matches. Both single words and predefined multiword phrases were identified using exact contiguous matching. Only word forms explicitly specified in the original appendix search formulas were counted; no stemming or lemmatization beyond those specified forms was applied. For each article, 3 dictionary-based metrics were calculated: (1) generative AI–associated terms per 1000 words, (2) control academic terms per 1000 words, and (3) the ratio of AI-associated to control terms.

### Statistical Analyses

Articles were analyzed according to whether they disclosed generative AI writing assistance. Articles without disclosed AI writing assistance served as the reference group. Descriptive statistics were summarized separately for articles with and without disclosed AI writing assistance. Categorical variables were summarized using counts and percentages and compared using Fisher exact test for 2 × 2 comparisons or the chi-square test for publication year. Continuous variables were summarized as medians with interquartile ranges and compared using the 2-sided Mann–Whitney *U* test. English-language–dominant affiliation fractions were summarized as mean ± standard deviation and compared using the Mann–Whitney *U* test because these fractions were bounded between 0 and 1.

Discussion-section readability metrics and predefined generative AI–associated lexical metrics were compared between articles with and without disclosed AI writing assistance using 2-sided Mann–Whitney *U* tests. For the full-cohort comparisons, between-group effects were summarized using Hodges–Lehmann location-shift estimates, calculated as values in articles with disclosed AI writing assistance minus those in articles without such disclosure. Their 95% confidence intervals (CIs) were obtained by inversion of the Wilcoxon rank-sum statistic using a tie-corrected normal approximation with a 0.5 continuity correction. Given the low prevalence of articles with disclosed AI writing assistance (n = 28), repeated stratified 1:5 resampling of articles without disclosed AI writing assistance was performed as a sensitivity analysis. Articles with disclosed AI writing assistance were retained as the exposed group, and comparison articles were sampled within strata defined by publication year and first-author English-language–dominant affiliation status. The procedure was repeated 1000 times. In each iteration, comparison articles were sampled at a 1:5 ratio relative to exposed articles within each stratum; sampling was performed without replacement when the available comparison pool was sufficient, with replacement permitted only if required. For each iteration, Hodges–Lehmann location-shift estimates for discussion-section readability metrics and predefined dictionary-based lexical metrics were calculated as values in articles with disclosed AI writing assistance minus those in articles without such disclosure. Mean Hodges–Lehmann location-shift estimates and 95% resampling intervals, defined by the 2.5th and 97.5th percentiles across iterations, were reported.

As secondary exploratory analyses restricted to articles without disclosed AI writing assistance, temporal trends in dictionary-based lexical metrics were evaluated across publication years. Yearly distributions were summarized as medians with interquartile ranges. Differences across publication years were assessed using the Kruskal–Wallis test. Year-only linear regression models were fitted with publication year as an ordinal variable; covariate-adjusted models additionally included first-author English-language–dominant affiliation status. Regression 95% CIs and *P* values were calculated using heteroskedasticity-consistent standard errors based on the type 3 covariance matrix estimator. For regression analysis, the AI-associated-to-control term ratio was natural log-transformed after adding 0.5 to both the numerator and denominator. These temporal analyses were interpreted descriptively and were not intended to classify individual articles as AI-assisted or non–AI-assisted.

To assess the influence of individual articles with disclosed AI writing assistance, leave-one-out sensitivity analyses were performed. The full-cohort comparisons were repeated 28 times, each time excluding 1 of the 28 articles with disclosed AI writing assistance while retaining all 605 articles without such disclosure. Hodges–Lehmann location-shift estimates and Holm-adjusted *P* values were recalculated in each iteration. The ranges of the resulting estimates and *P* values, preservation of the full-sample effect direction, and the number of iterations retaining statistical significance were summarized.

To address multiplicity, Holm adjustment was applied separately within the 3 readability indices, 5 underlying text components, and 3 lexical metrics. For temporal analyses, adjustment was applied across the 3 lexical outcomes separately for each analysis type (Kruskal–Wallis, year-only regression, and covariate-adjusted regression). Stratified resampling was used to summarize empirical distributions of effect estimates and was not treated as an additional multiplicity family. Holm adjustment was recalculated separately for the readability and lexical metrics within each leave-one-out iteration. All statistical tests were 2-sided, and *P* < 0.05 was considered statistically significant after multiplicity adjustment where applicable. Analyses were conducted in Python 3.9.23 using pandas 2.3.1, NumPy 2.0.2, SciPy 1.13.1, textstat 0.7.13, and statsmodels 0.14.5.

## Results

A total of 633 original research articles published in *Ophthalmology Science* during the study period were included in the analysis after exclusion of 1 article without extractable discussion-section text. Of these, 28 articles (4.4%) disclosed generative AI assistance for manuscript preparation or writing-related purposes. Characteristics of articles with and without disclosed AI writing assistance are summarized in Table 1. English-language–dominant affiliation fractions were significantly lower among AI-disclosed articles for both first authors (*P* = 0.008) and corresponding authors (*P* = 0.012). Intermediate values between 0 and 1 occurred in 2 of 28 (7.1%) AI-disclosed and 37 of 605 (6.1%) articles without disclosed AI writing assistance for first-author fractions and in 1 of 28 (3.6%) and 22 of 604 (3.6%) articles, respectively, for corresponding-author fractions. Publication year distribution differed between groups (*P* = 0.022), whereas the number of authors did not (*P* = 0.624).

**Table 1:** Characteristics of Articles by Disclosed Generative AI Writing Assistance

Variable	Disclosed AI Writing Assistance (n = 28)	No Disclosed AI Writing Assistance (n = 605)	*P* Value
English-language–dominant country affiliation of first author (fraction, mean ± SD)	0.37 ± 0.47	0.60 ± 0.48	0.008∗
English-language–dominant country affiliation of corresponding author (fraction, mean ± SD)	0.38 ± 0.48	0.61 ± 0.48	0.012∗
Publication year			
2024	4 (14.3%)	168 (27.8%)	0.022∗
2025	8 (28.6%)	242 (40.0%)	
2026	16 (57.1%)	195 (32.2%)	
Number of authors (median, IQR)	8.5 (7.0–11.0)	8.0 (6.0–12.0)	0.624

AI = artificial intelligence; IQR = interquartile range; SD = standard deviation.

Values are presented as mean ± SD for English-language–dominant affiliation fractions, n (%) for publication year, and median (IQR) for number of authors.

The reference group included articles without disclosed AI writing assistance, including articles with no AI declaration, articles stating that AI was not used for manuscript preparation, and articles reporting generative AI use solely as part of the research methodology.

English-language–dominant countries were defined as the United States, United Kingdom, Canada, Australia, New Zealand, and Ireland.

Affiliation fractions were calculated using fractional weighting across cofirst or cocorresponding authors and multiple affiliations, yielding article-level values from 0 to 1.

*P* values were calculated using Fisher exact test for 2 × 2 categorical comparisons, the chi-square test for publication year, and the Mann–Whitney U test for continuous variables.

∗ *P* < 0.05.

Disclosure texts and reported AI tools for articles classified as having disclosed generative AI writing assistance are provided in Table S1 (available at www.ophthalmologyscience.org). All included disclosure statements indicated that the authors took full responsibility for the content of the publication.

### Discussion Readability

In the full-cohort analysis, articles with disclosed AI writing assistance had lower FRE (Hodges–Lehmann location shift, –8.52; 95% CI, –13.13 to –3.99; Holm-adjusted *P* < 0.001), higher FKGL (1.11; 95% CI, 0.32–1.94; Holm-adjusted *P* = 0.013), and higher SMOG scores (0.70; 95% CI, 0.09–1.38; Holm-adjusted *P* = 0.027) than articles without such disclosure (Table 2; Fig 1A). Across 1000 stratified 1:5 resampling iterations, the mean Hodges–Lehmann location shifts were –7.35 for FRE (95% resampling interval, –8.90 to –5.83), 1.06 for FKGL (95% resampling interval, 0.80–1.32), and 0.66 for SMOG (95% resampling interval, 0.47–0.86), showing consistent effect directions across alternative comparison samples (Fig 1B).

**Table 2:** Discussion-Section Readability and Lexical Metrics by Disclosed Generative AI Writing Assistance

Section	Outcome	Disclosed AI Writing Assistance (n = 28), Median (IQR)	No Disclosed AI Writing Assistance (n = 605), Median (IQR)	Hodges–Lehmann Location Shift (95% CI)	Holm-Adjusted *P* Value
Readability indices	Flesch Reading Ease	11.97 (7.01–20.99)	21.59 (14.21–27.53)	–8.52 (–13.13–3.99)	<0.001∗
	Flesch–Kincaid Grade Level	17.15 (15.70–18.31)	16.00 (14.89–17.30)	1.11 (0.32–1.94)	0.013∗
	SMOG index	17.82 (16.71–18.75)	17.12 (16.11–18.16)	0.70 (0.09–1.38)	0.027∗
Underlying components	Discussion word count	1274.50 (988.50–1610.75)	1210.00 (998.00–1515.00)	33.00 (–121.00 to 192.00)	1.000
	Mean sentence length, words/sentence	22.56 (21.10–24.32)	22.63 (20.62–25.10)	–0.29 (–1.49 to 0.89)	1.000
	Mean syllables per word	2.05 (1.91–2.09)	1.91 (1.84–1.99)	0.11 (0.06–0.16)	<0.001∗
	Polysyllabic words (≥3 syllables), count	374.00 (263.25–473.00)	314.00 (247.00–391.00)	54.00 (5.00–105.00)	0.094
	Polysyllabic words per 100 words	29.00 (25.98–32.24)	26.13 (23.51–28.66)	3.10 (1.41–4.90)	0.002∗
Lexical metrics	AI-associated terms per 1000 words	22.38 (16.69–29.85)	15.71 (10.75–22.64)	6.38 (2.76–9.88)	0.003∗
	Control academic terms per 1000 words	25.64 (20.44–29.68)	24.72 (19.34–31.80)	–0.38 (–3.84 to 3.06)	0.844
	AI-associated-to-control term ratio	0.91 (0.69–1.19)	0.63 (0.41–0.97)	0.26 (0.10–0.41)	0.004∗

AI = artificial intelligence; CI = confidence interval; FKGL = Flesch–Kincaid Grade Level; FRE = Flesch Reading Ease; IQR = interquartile range; SMOG = Simple Measure of Gobbledygook.

Values are presented as median (IQR). Hodges–Lehmann location shifts and their 95% CIs were calculated as values in articles with disclosed AI writing assistance minus those in articles without disclosed AI writing assistance. Accordingly, negative FRE shifts and positive FKGL or SMOG shifts indicate lower readability among articles with disclosed AI writing assistance.

The Hodges–Lehmann estimate is the median of all pairwise between-group differences and represents a location shift rather than a difference between sample medians. Two-sided *P* values were obtained using the Mann–Whitney *U* test. The 95% CIs were obtained by inversion of the Wilcoxon rank-sum statistic using a tie-corrected normal approximation with a 0.5 continuity correction.

Holm adjustment was applied separately within the 3 readability indices, 5 underlying text components, and 3 lexical metrics, using unrounded *P* values. Polysyllabic words were defined as words with ≥3 syllables.

∗ *P* < 0.05 after Holm adjustment.

**Figure 1:**
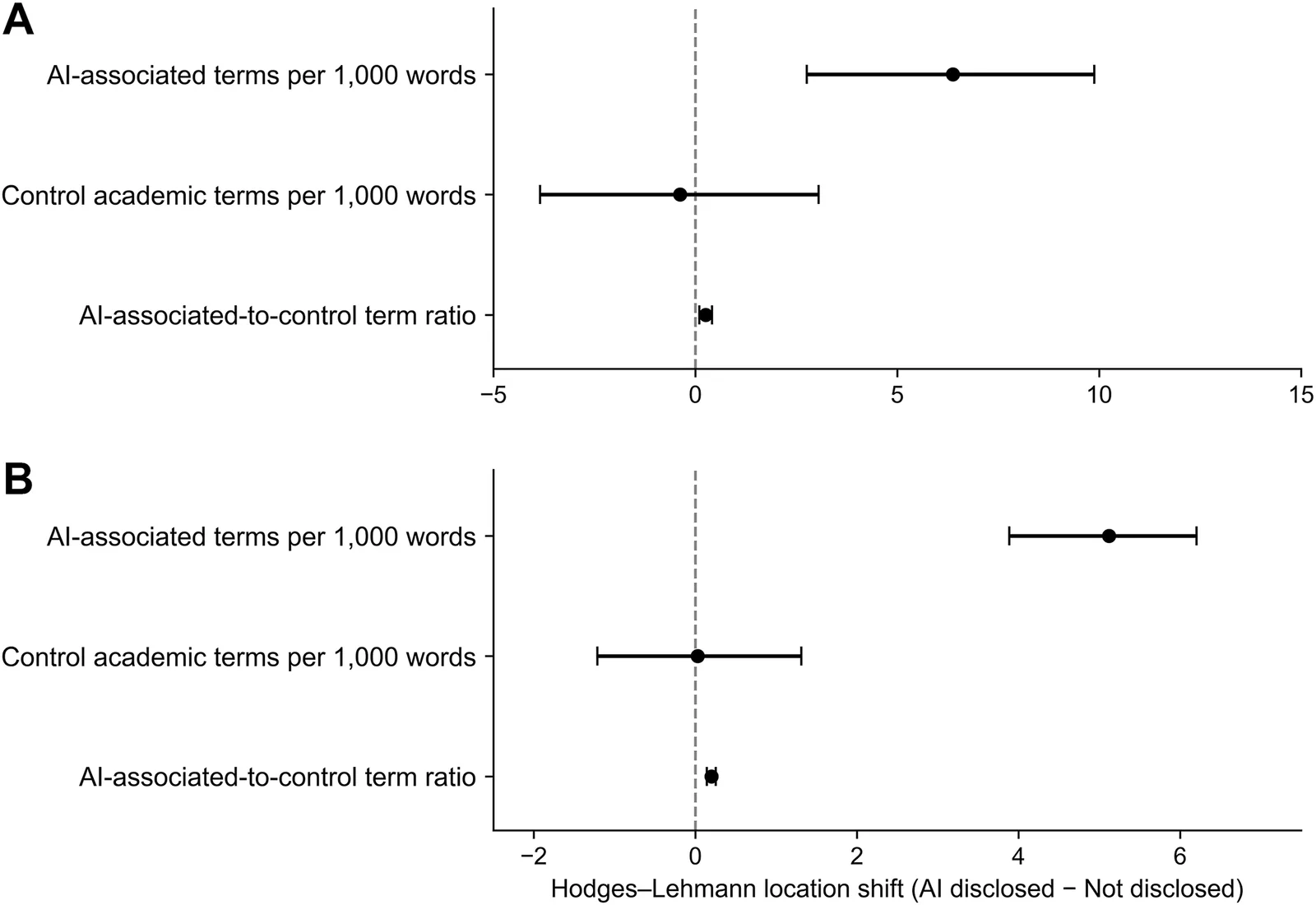
Discussion-section readability differences by disclosed generative AI writing assistance. **A,** Full-cohort Hodges–Lehmann location-shift estimates and 95% confidence intervals. **B,** The mean Hodges–Lehmann location-shift estimates across 1000 stratified 1:5 resampling iterations; horizontal bars indicate the 2.5th and 97.5th percentiles across iterations. Articles without disclosed AI writing assistance were sampled within strata defined by publication year and first-author English-language–dominant affiliation status. Effects were calculated as values in articles with disclosed AI writing assistance minus those in articles without such disclosure. Negative Flesch Reading Ease shifts and positive Flesch–Kincaid Grade Level or Simple Measure of Gobbledygook shifts indicate lower readability among articles with disclosed AI writing assistance. AI = artificial intelligence.

Analysis of the underlying components suggested that the observed readability differences were more closely related to lexical complexity than to sentence length (Table 2). Articles with disclosed AI writing assistance had higher mean syllables per word (Holm-adjusted *P* < 0.001) and more polysyllabic words per 100 words (Holm-adjusted *P* = 0.002), whereas neither discussion word count nor mean sentence length differed between groups (Holm-adjusted *P* = 1.000 for both). Although the absolute polysyllabic-word count showed a positive location-shift estimate (54 words; 95% CI, 5–105), this comparison did not meet the Holm-adjusted significance threshold (*P* = 0.094).

In the 28 leave-one-out iterations, the effect directions were preserved in all iterations for FRE, FKGL, and SMOG. The Hodges–Lehmann location shifts ranged from –9.31 to –7.72 for FRE, 0.99 to 1.23 for FKGL, and 0.60 to 0.79 for SMOG. Holm-adjusted *P* values were <0.05 in all 28 iterations for FRE and FKGL and in 22 of 28 iterations for SMOG (Table S2, available at www.ophthalmologyscience.org).

### Dictionary-Based Lexical Analysis

In the full-cohort analysis, articles with disclosed AI writing assistance had a higher density of predefined AI-associated terms (Hodges–Lehmann location shift, 6.38 terms per 1000 words; 95% CI, 2.76–9.88; Holm-adjusted *P* = 0.003) and a higher AI-associated-to-control term ratio (0.26; 95% CI, 0.10–0.41; Holm-adjusted *P* = 0.004) than articles without such disclosure. Control academic term density did not differ between groups (–0.38 terms per 1000 words; 95% CI, –3.84 to 3.06; Holm-adjusted *P* = 0.844) (Table 2; Fig 2A). Across 1000 stratified 1:5 resampling iterations, the mean Hodges–Lehmann location shifts were 5.13 terms per 1000 words for AI-associated term density (95% resampling interval, 3.88–6.21), 0.03 terms per 1000 words for control academic term density (95% resampling interval, –1.21 to 1.31), and 0.21 for the AI-associated-to-control term ratio (95% resampling interval, 0.14–0.25) (Fig 2B).

**Figure 2:**
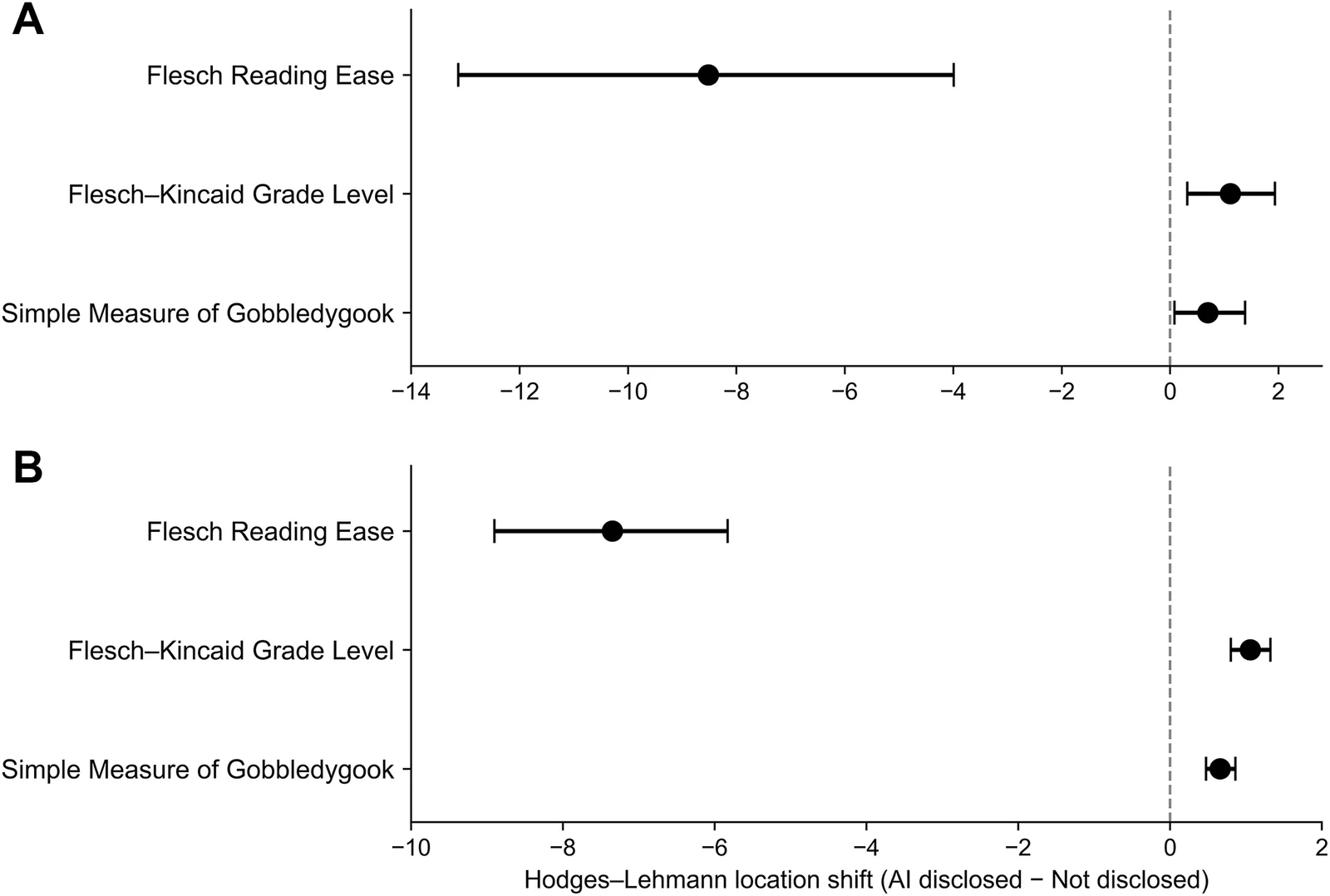
Discussion-section dictionary-based lexical differences by disclosed generative AI writing assistance. **A,** Full-cohort Hodges–Lehmann location-shift estimates and 95% confidence intervals. **B,** The mean Hodges–Lehmann location-shift estimates across 1000 stratified 1:5 resampling iterations; horizontal bars indicate the 2.5th and 97.5th percentiles across iterations. Articles without disclosed AI writing assistance were sampled within strata defined by publication year and first-author English-language–dominant affiliation status. Effects were calculated as values in articles with disclosed AI writing assistance minus those in articles without such disclosure. AI = artificial intelligence.

In the 28 leave-one-out iterations, the full-sample effect directions were preserved in all iterations for AI-associated term density and the AI-associated-to-control term ratio and in 22 of 28 iterations for control academic term density. The Hodges–Lehmann location shifts ranged from 5.83 to 6.98 terms per 1000 words for AI-associated term density, –0.86 to 0.20 terms per 1000 words for control academic term density, and 0.24 to 0.28 for the term ratio. Holm-adjusted *P* values were <0.05 in all 28 iterations for AI-associated term density and the term ratio and in none of the 28 iterations for control academic term density (Table S2).

### Temporal Trends among Articles without Disclosed AI Writing Assistance

Among articles without disclosed AI writing assistance, predefined generative AI–associated term density and the AI-associated-to-control term ratio increased over publication years (Holm-adjusted *P* < 0.001 for both), whereas control academic term density did not (Holm-adjusted *P* = 0.120) (Table S3, available at www.ophthalmologyscience.org). In regression models adjusted for first-author English-language–dominant affiliation status, the year effects remained significant for AI-associated term density and the term ratio (Holm-adjusted *P* < 0.001 for both) but not for control academic term density (Holm-adjusted *P* = 0.142) (Table S3).

## Discussion

This study examined linguistic characteristics in the discussion sections of original research articles published in *Ophthalmology Science*, using disclosed generative AI writing assistance as an observable disclosure-based classification rather than as verification of actual AI use. By anchoring the analysis to explicit statements of writing-related AI assistance rather than temporal proxies or automated AI-detection algorithms, this design provides an observational framework for examining linguistic patterns associated with AI writing-assistance disclosure. In the expanded corpus, disclosed AI writing assistance remained uncommon but was more frequently observed among authors affiliated with non–English-language–dominant countries. Articles with disclosed AI writing assistance showed lower discussion-section readability, higher estimated reading grade levels, and greater density of predefined generative AI–associated terms. In contrast, no detectable difference was observed in the selected control academic terms. In the secondary temporal analysis restricted to articles without disclosed AI writing assistance, AI-associated term density increased over publication years, whereas control academic terms did not show a corresponding increase. Together, these findings indicate that disclosed AI writing assistance was associated with measurable differences in discussion-section readability and predefined AI-associated lexical patterns, while temporal trends among nondisclosing articles were consistent with corpus-level diffusion of AI-associated vocabulary.

### Adoption Patterns of Disclosed Generative AI Use

In the present study, disclosed generative AI writing assistance was uncommon, observed in 28 of 633 original research articles (4.4%). During the study period (January 2024 through May 2026), publisher guidance on generative AI use in manuscript preparation had already been issued and emphasized transparent reporting when applicable. Although recent advances in LLMs have stimulated the incorporation of generative AI tools into scientific writing and editing workflows,^6^^,^^7^ the limited frequency of disclosure in this journal suggests that disclosed generative AI assistance may still have been in an early, evolving phase during the observation window. A recent large-scale analysis of more than 164 000 scientific publications^14^ similarly highlighted a substantial transparency gap: among approximately 75 000 papers published since 2023, only 76 (≈0.1%) explicitly disclosed AI use. Although that study examined full-text disclosures across multiple disciplines rather than journal-specific declaration statements, its findings reinforce the possibility that formal disclosure may substantially underestimate actual generative AI use in scientific writing. Importantly, the presence or absence of a disclosure statement cannot be used to infer the actual prevalence of generative AI use, as disclosures are self-reported and unmeasured use without declaration cannot be evaluated within the current design. Therefore, the present findings should be interpreted as comparisons based on disclosed LLM involvement rather than estimates of overall use.

This study also observed that articles with disclosed AI writing assistance had lower English-language–dominant affiliation fractions for both first authors and corresponding authors. This pattern may be consistent with prior literature describing the substantial time and financial burdens associated with publishing in English for researchers working in non-English contexts, and with the broader argument that AI-based language assistance could help mitigate linguistic inequities in scientific communication.^15^, ^16^, ^17^ However, institutional country affiliation is an imperfect proxy for individual language proficiency, writing responsibility, or access to professional editorial support, and residual confounding by these factors cannot be excluded. The near-dichotomous distribution of the affiliation fractions also limited the information available for continuous adjustment. Although direct identification of the author or authors responsible for drafting each manuscript would have been preferable, available contributorship information does not consistently distinguish manuscript drafting from critical revision or broader manuscript preparation. Accordingly, this observation should be interpreted cautiously.

### Readability and Prose Structure Associated with Disclosed AI Writing Assistance

Articles disclosing generative AI use showed lower FRE and higher FKGL and SMOG scores in the discussion section, indicating lower formula-based readability and a higher estimated reading grade level. Although language-support tools are generally expected to improve clarity and readability,^6^^,^^18^ the present findings were more consistent with greater lexical complexity than with simplification. One possible explanation is that generative AI writing assistance may favor syllabically complex academic vocabulary. Although SMOG estimates remained positive across all leave-one-out iterations, statistical evidence was somewhat less stable for SMOG, a metric based on polysyllabic words per sentence, than for FRE and FKGL, both of which combine mean syllables per word with mean sentence length. The component analysis also showed higher mean syllables per word and a higher proportion of polysyllabic words in articles with disclosed AI writing assistance, but no detectable difference in mean sentence length. These findings suggest that the observed readability differences primarily reflected greater lexical complexity rather than longer sentences. However, readability formulas capture only limited structural features and do not directly assess writing quality, coherence, understandability, conceptual clarity, or scientific quality. Moreover, because pre-assistance manuscripts were not available, the observed differences cannot be attributed causally to LLM output itself. Notably, the same direction of readability differences persisted after stratified resampling by publication year and first-author English-language–dominant affiliation status. This finding suggests that the observed readability pattern was not fully explained by publication timing or first-author institutional language context alone, and may reflect prose-level patterns associated with disclosed AI writing assistance.

### AI-Associated Lexical Patterns and Temporal Diffusion

One of the important findings of this study was that articles with disclosed generative AI writing assistance showed a higher density of predefined generative AI–associated terms and a higher ratio of AI-associated to control terms in the discussion section. In contrast, no detectable group difference was observed in the selected control academic terms. Together with the higher AI-associated-to-control term ratio, this pattern suggests a relative predominance of the predefined AI-associated term set rather than a uniform difference across both dictionaries. Prior studies have identified words frequently appearing in LLM-generated text^19^^,^^20^ or inferred AI influence through temporal analyses of vocabulary trends following the emergence of ChatGPT.^11^^,^^12^^,^^21^^,^^22^ However, such approaches are generally not anchored to article-level disclosure of AI writing assistance, and therefore may have difficulty distinguishing AI-associated wording from broader temporal changes in academic language. In contrast, the present study used disclosed AI writing assistance as an observable anchor and compared articles with and without such disclosure within a single ophthalmology journal. This design provides empirical evidence that predefined AI-associated vocabulary was more frequent among articles with disclosed AI writing assistance, rather than merely reflecting time-dependent shifts in scientific language. However, because the 2 term sets were not matched for baseline frequency, topic neutrality, or part-of-speech distribution, this contrast is limited to the predefined dictionaries and does not by itself establish an AI-specific lexical signal.

In the secondary temporal analyses restricted to articles without disclosed AI writing assistance, AI-associated term density also increased over publication years, whereas control academic term density did not show a corresponding temporal increase. One possible interpretation is that AI-associated lexical patterns are diffusing across the journal corpus as part of broader lexical change within the scholarly writing ecosystem, including articles without formal writing-assistance disclosure. Linguistic innovations and borrowed or discipline-specific expressions can spread through professional communities via repeated exposure to published texts, editorial practices, peer review, author templates, and routine scholarly communication, without requiring each adopter to have direct exposure to the original source-language or source-context environment.^23^^,^^24^ Accordingly, articles without disclosed AI writing assistance should not be regarded as an uncontaminated AI-unexposed control group, but as a comparison group situated within the same evolving linguistic environment. At the same time, these results should be interpreted cautiously. The presence or absence of disclosed AI writing assistance does not verify actual AI use at the article level, and temporal increases among articles without disclosed AI writing assistance should not be interpreted as evidence of undisclosed AI use in individual manuscripts. Authors who disclose AI writing assistance may also differ in baseline writing style, language background, editorial workflows, or willingness to disclose AI use. Nevertheless, the combination of a higher density of predefined AI-associated terms among articles with disclosed AI writing assistance, a temporal increase in these terms among articles without disclosed AI writing assistance, and the absence of corresponding detectable group differences or temporal increases in the selected control academic terms suggests that the observed lexical pattern was not fully explained by publication timing or by a uniform change across the 2 predefined term sets.

### Strengths, Limitations, and Conclusion

Several strengths and limitations should be noted. A key strength of this study is its disclosure-anchored design, which enables a direct comparison of published articles with acknowledged LLM involvement versus those without disclosure within the same journal and time window. In addition, the study focused a priori on the discussion section and evaluated complementary dimensions of writing, including readability and predefined dictionary-based lexical metrics with control academic terms. The use of fractional first-author and corresponding-author English-language–dominant affiliation measures, together with stratified resampling by publication year and first-author English-language–dominant affiliation status, helped assess whether the observed patterns were robust to differences in publication timing and institutional language context.

Several limitations should be noted. First, LLM involvement was defined using formal disclosure statements, which are self-reported and do not necessarily reflect the true prevalence or extent of generative AI use. Therefore, articles without disclosed AI writing assistance should not be interpreted as verified non–AI-assisted manuscripts. Second, the number of articles with disclosed AI writing assistance was limited. Although a stratified resampling approach and leave-one-out analyses were applied to assess robustness, statistical precision remains constrained. The directions and magnitudes of the observed readability and AI-associated lexical effects were generally stable to removal of any single disclosed article, although the Holm-adjusted *P* value for SMOG remained <0.05 in 22 of 28 iterations. Thus, the magnitude and statistical significance of smaller effects may change as additional disclosed articles become available. Third, the study was restricted to a single ophthalmology journal during a defined time period. Patterns may differ across disciplines or as LLM adoption evolves. Fourth, readability indices may be influenced by subspecialty and article topic because polysyllabic ophthalmic terminology is counted as lexical complexity. Because subspecialty and topic were not modeled and no ophthalmology-specific syllable dictionary or manual term correction was used, differences in technical terminology between disclosure groups, together with possible syllable-counting errors for specialty terms, may have contributed to the observed readability differences. Finally, no independent measure of writing quality, coherence, or understandability was assessed. The readability and lexical metrics used in this study capture surface textual features and predefined lexical categories rather than conceptual clarity, scientific accuracy, or overall manuscript quality. These findings should therefore be interpreted as disclosure-associated linguistic patterns rather than definitive evidence of AI-generated text or causal effects of AI writing assistance.

In conclusion, articles with disclosed generative AI writing assistance showed measurable differences in discussion-section language, including lower formula-based readability, higher estimated reading grade levels, and a higher density of predefined generative AI–associated terms. Secondary temporal analyses were consistent with diffusion of AI-associated lexical patterns among articles without disclosed AI writing assistance. As generative AI becomes increasingly integrated into scientific writing workflows, generative AI writing assistance may be understood not only as a technical aid for language editing but also as a contextual factor associated with evolving patterns of scholarly language.

## Declaration of Generative AI and AI-Assisted Technologies in the Writing Process

During the preparation of this work, the author (T.I.) used OpenAI GPT-5 Pro to improve readability and language. After using this tool, the author (T.I.) reviewed and edited the content as needed and takes full responsibility for the content of the publication. Generative AI tools were used solely for language editing and were not used to analyze or process copyrighted journal content.
