# Supplementary material for: Lexical Diffusion: Linguistic Characteristics of Ophthalmology Science Articles with Disclosed Artificial Intelligence-Assisted Writing
Source: Ophthalmol Sci. 2026 Sep 21;6(11):101418. doi: 10.1016/j.xops.2026.101418 (PMC13694793; doi:10.1016/j.xops.2026.101418)
Supplement: Supplementary file 1 [file mmc1.pdf]

Supplementary Table S1. Generative AI Writing-Assistance Disclosure Statements and Reported AI Tools

Generative AI Disclosure Statement (verbatim)	Reported AI Tool
During the preparation of this work, the author used Chat-GPT in order to improve readability and language. After using this tool, the authors reviewed and edited the content to ensure all statements were correct. The authors take full responsibility for the content of the publication.	Chat-GPT
During the preparation of this work, the authors used GPT-3.5 in order to improve language. After using this tool, the authors reviewed and edited the content as needed and take full responsibility for the content of the publication.	GPT-3.5
During the preparation of this work, the author(s) used Google Translate in order to translate from Swedish to English. In addition, this article was edited by Elsevier Language Editing Service. After using this tool/service, the author(s) reviewed and edited the content as needed and took(s) full responsibility for the content of the publication.	Google Translate
During the preparation of this work the authors used OpenAI's large language model for linguistic corrections to enhance the clarity of this manuscript. After using this tool, the authors reviewed and edited the content as needed and take full responsibility for the content of the publication.	OpenAI's large language model
During the preparation of this work, the authors used ChatGPT4 in order to improve the manuscript’s language and readability. After using this tool, the authors reviewed and edited the content as needed and take full responsibility for the content of the publication.	ChatGPT4
During the preparation of this work, the authors used ChatGPT 4o to provide feedback on grammar and syntax. After using this tool/service, the authors reviewed and edited the content as needed and take full responsibility for the content of the publication.	ChatGPT 4o

Supplementary Table S1. Generative AI Writing-Assistance Disclosure Statements and Reported AI Tools

Generative AI Disclosure Statement (verbatim)	Reported AI Tool
During the preparation of this manuscript, the authors used Claude 3.5 Sonnet (Anthropic) to help improve the readability and language of specific sections of the manuscript. The artificial intelligence tool was not used for data analysis or scientific interpretation. After using this tool, the authors thoroughly reviewed and edited the content as needed and take full responsibility for the content of the published article.	Claude 3.5 Sonnet (Anthropic)
During the preparation of this work, the authors used Kimi AI (https://kimi.moonshot.cn/) to enhance the readability and coherence of the manuscript, optimize vocabulary, and ensure clear presentation of complex ideas. After using this tool, the authors reviewed and edited the content thoroughly. The authors take full responsibility for the content of the publication.	Kimi AI (https://kimi.moonshot.cn/)
During the preparation of this work the authors used ChatGPT, an AI-based language model developed by OpenAI, in order to edit the English language of this manuscript. After using this tool/service, the authors reviewed and edited the content as needed and take full responsibility for the content of the publication.	ChatGPT
During the preparation of this manuscript, the authors used Claude 3.7 Sonnet (Anthropic) to help improve the readability and language of specific sections of the manuscript. The AI tool was not used for data analysis or scientific interpretation. After using this tool, the authors thoroughly reviewed and edited the content as needed and take full responsibility for the content of the published article.	Claude 3.7 Sonnet (Anthropic)
During the preparation of this work, the authors used Grammarly to correct the grammar. After using this tool/service, the authors reviewed and edited the content as needed and take full responsibility for the content of the publication.	Grammarly

Supplementary Table S1. Generative AI Writing-Assistance Disclosure Statements and Reported AI Tools

Generative AI Disclosure Statement (verbatim)	Reported AI Tool
During the preparation of this work the author(s) used ChatGPT for grammar and spelling check. After using this tool/service, the authors reviewed and edited the content as needed and take full responsibility for the content of the publication.	ChatGPT
During the preparation of this work, the authors used chatGPT4.0 to improve readability and language of the manuscript. After using this tool/service, the authors reviewed and edited the content as needed and took full responsibility for the content of the publication.	chatGPT4.0
During the preparation of this work, the authors used ChatGPT 4o in order to improve readability and language. After using this tool, the authors reviewed and edited the content as needed and take full responsibility for the content of the publication.	ChatGPT 4o
During the preparation of this work, the author(s) used ChatGPT version 5 for grammar and spelling checks. After using this tool/service, the authors reviewed and edited the content as needed and take full responsibility for the content of the publication.	ChatGPT version 5
During the preparation of this work the authors used ChatGPT-4o in order to improve readability and language of the manuscript. After using this tool, the authors reviewed and edited the content as needed and take full responsibility for the content of the published article.	ChatGPT-4o
During the preparation of this work, the authors used OpenAI GPT-4 in order to improve readability and language. After using this tool/service, the authors reviewed and edited the content as needed and take full responsibility for the content of the publication.	OpenAI GPT-4
During the preparation of this work, the authors used OpenAI GPT-5 Pro, in August 2025, to improve readability and language. After using this tool, the authors reviewed and edited the content as needed and take full responsibility for the content of the publication.	OpenAI GPT-5 Pro

Supplementary Table S1. Generative AI Writing-Assistance Disclosure Statements and Reported AI Tools

Generative AI Disclosure Statement (verbatim)	Reported AI Tool
During the preparation of this work, the authors used ChatGPT in order to improve scientific writing. After using this tool, the authors reviewed and edited the content as needed and take full responsibility for the content of the publication.	ChatGPT
During the preparation of this work the authors used ChatGPT in order to improve the language and readability. After using this tool, the authors reviewed and edited the content as needed and take full responsibility for the content of the publication.	ChatGPT
During the preparation of this manuscript, the author (M.K.) used ChatGPT-4o mini to assist with translating the original Japanese draft into English. Subsequently, the authors reviewed and revised the content as needed and took full responsibility for the content of the published article.	ChatGPT-4o mini
During the preparation of this work the authors used ChatGPT in order to improve language and readability. After using this tool/service, the authors reviewed and edited the content as needed and take full responsibility for the content of the publication.	ChatGPT
During the preparation of this work the authors used ChatGPT in order to improve clarity of language. The tool was not used for data generation, analysis, or interpretation. After using this tool, the authors reviewed and edited the content as needed and take full responsibility for the content of the published article.	ChatGPT
During the preparation of this work, the authors used ChatGPT for minor language editing. After using this tool/service, the authors reviewed and edited the content as needed and take full responsibility for the content of the published article.	ChatGPT

Supplementary Table S1. Generative AI Writing-Assistance Disclosure Statements and Reported AI Tools

Generative AI Disclosure Statement (verbatim)	Reported AI Tool
During the preparation of this work, the author(s) used ChatGPT in order to improve readability of the manuscript. After using this tool/service, the author(s) reviewed and edited the content as needed and take(s) full responsibility for the content of the publication.	ChatGPT
During the preparation of this work the author(s) used Microsoft Copilot in order to refine the grammar and writing style. After using this tool/service, the author(s) reviewed and edited the content as needed and take(s) full responsibility for the content of the publication.	Microsoft Copilot
During the preparation of this work the author(s) used ChatGPT in order to improve the clarity and readability of the manuscript. After using this tool/service, the author(s) reviewed and edited the content as needed and take(s) full responsibility for the content of the published article.	ChatGPT
During the preparation of this work the authors used Microsoft Copilot in order to improve language and readability. After using this tool, the authors reviewed and edited the content as needed and take full responsibility for the content of the publication.	Microsoft Copilot

Disclosure statements are reproduced verbatim from the published articles. AI tools were identified directly from the disclosure text without modification or normalization. No additional interpretation or categorization was applied.
AI = artificial intelligence.
