# Supplementary material for: Lexical Diffusion: Linguistic Characteristics of Ophthalmology Science Articles with Disclosed Artificial Intelligence-Assisted Writing
Source: Ophthalmol Sci. 2026 Sep 21;6(11):101418. doi: 10.1016/j.xops.2026.101418 (PMC13694793; doi:10.1016/j.xops.2026.101418)
Supplement: Supplementary file 2 [file mmc2.pdf]

Supplementary Table S2. Leave-one-out sensitivity analyses of discussion-section readability and lexical metrics

Domain	Outcome	Full-sample HL location shift (95% CI)	Holm-adjusted *P* value	Leave-one-out HL location-shift range	Leave-one-out Holm-adjusted *P* value range	Same sign as full-sample HL estimate, n/N	Holm-adjusted *P* < 0.05, n/N
Readability indices	Flesch Reading Ease	−8.52 (−13.13 to −3.99)	<0.001*	−9.31 to −7.72	<0.001 to 0.002	28/28	28/28
	Flesch−Kincaid Grade Level	1.11 (0.32 to 1.94)	0.013*	0.99 to 1.23	0.004 to 0.029	28/28	28/28
	SMOG Index	0.70 (0.09 to 1.38)	0.027*	0.60 to 0.79	0.011 to 0.054	28/28	22/28
Lexical metrics	AI-associated terms per 1,000 words	6.38 (2.76 to 9.88)	0.003*	5.83 to 6.98	<0.001 to 0.007	28/28	28/28
	Control academic terms per 1,000 words	−0.38 (−3.84 to 3.06)	0.844	−0.86 to 0.20	0.639 to 0.988	22/28	0/28
	AI-associated-to-control term ratio	0.26 (0.10 to 0.41)	0.004*	0.24 to 0.28	0.001 to 0.011	28/28	28/28

The full-sample analysis included 28 articles with disclosed generative AI writing assistance and 605 articles without such disclosure. Each of the 28 leave-one-out iterations excluded 1 article with disclosed generative AI writing assistance, leaving 27 articles in that group; the 605-article comparison group was unchanged.

Hodges–Lehmann location shifts were calculated as values in articles with disclosed AI writing assistance minus those in articles without disclosed AI writing assistance. The full-sample Hodges–Lehmann estimate is the median of all pairwise between-group differences. Its 95% CI was obtained by inversion of the Wilcoxon rank-sum statistic using a tie-corrected normal approximation with a 0.5 continuity correction. Negative FRE shifts and positive FKGL or SMOG shifts indicate lower readability among articles with disclosed AI writing assistance.

Leave-one-out ranges show the minimum and maximum values across the 28 iterations and are not CIs. “Same sign as full-sample HL estimate, n/N” indicates the number of leave-one-out iterations in which the Hodges–Lehmann estimate had the same sign as the full-sample estimate. This measure describes sign consistency only and does not incorporate effect magnitude, confidence intervals, or statistical significance. “Holm-adjusted *P* <0.05, n/N” indicates the number of iterations with a Holm-adjusted *P* value <0.05.

Holm adjustment was applied separately within the three readability indices and the three lexical metrics in the full sample and within each leave-one-out iteration, using unrounded *P* values. The leave-one-out analyses repeated the same endpoints as sensitivity analyses and were not treated as additional multiplicity families.

**P* <0.05 after Holm adjustment in the full-sample analysis; asterisks are not applied to range columns.

AI = artificial intelligence; CI = confidence interval; FKGL = Flesch–Kincaid Grade Level; FRE = Flesch Reading Ease; HL = Hodges–Lehmann; SMOG = Simple Measure of Gobbledygook.
