# Supplementary material for: Lexical Diffusion: Linguistic Characteristics of Ophthalmology Science Articles with Disclosed Artificial Intelligence-Assisted Writing
Source: Ophthalmol Sci. 2026 Sep 21;6(11):101418. doi: 10.1016/j.xops.2026.101418 (PMC13694793; doi:10.1016/j.xops.2026.101418)
Supplement: Supplementary file 3 [file mmc3.pdf]

Supplementary Table S3. Temporal trends in discussion-section dictionary-based lexical metrics among articles without disclosed generative AI writing assistance

Outcome	2024, median (IQR)	2025, median (IQR)	2026, median (IQR)	Kruskal−Wallis Holm-adjusted *P*	Year-only regression β (95% CI)	Year-only regression Holm-adjusted *P*	Covariate-adjusted regression β (95% CI)	Covariate-adjusted regression Holm-adjusted *P*
AI-associated terms per 1,000 words	12.50 (8.25−17.23)	16.47 (11.25−25.48)	17.96 (13.00−24.48)	<0.001*	2.75 (1.95 to 3.55)	<0.001*	2.55 (1.73 to 3.36)	<0.001*
Control academic terms per 1,000 words	24.77 (20.22−31.63)	25.63 (19.74−32.42)	23.53 (18.19−29.73)	0.120	−0.83 (−1.83 to 0.18)	0.106	−0.77 (−1.81 to 0.26)	0.142
AI-associated-to-control term ratio	0.48 (0.34−0.70)	0.63 (0.42−1.05)	0.76 (0.54−1.07)	<0.001*	0.217 (0.152 to 0.281)	<0.001*	0.206 (0.139 to 0.274)	<0.001*

Analyses were restricted to the 605 articles without disclosed generative AI writing assistance. Year-specific values are presented as median (IQR). Differences across publication years were assessed using the Kruskal–Wallis test.

Year-only models included publication year only; covariate-adjusted models additionally included first-author English-language–dominant affiliation status, defined using a weighted affiliation fraction threshold of ≥0.5. Regression 95% CIs and *P* values were calculated using heteroskedasticity-consistent standard errors based on the type 3 covariance matrix estimator.

For the AI-associated-to-control term ratio, year-specific summaries show the untransformed ratio, whereas regression analyses used the natural-log–transformed ratio after adding 0.5 to both the numerator and denominator.

Holm adjustment was applied separately across the three lexical outcomes within each analysis type—the Kruskal–Wallis test, year-only regression, and covariate-adjusted regression—using unrounded *P* values. All displayed *P* values are Holm-adjusted for multiplicity.

**P* <0.05 after Holm adjustment.

AI = artificial intelligence; β = regression coefficient; CI = confidence interval; IQR = interquartile range.
